# Association Between Recurring Headache and Selected Women's Health Issues Among U.S. Navy and Marine Corps Women: Cross-Sectional Results of the Annual Periodic Health Assessment, 2021

**Published:** 2025-05-01

**Authors:** James K. Romine, Amber L. Dougherty, Mary J. Hessert, Andrew J. MacGregor

**Affiliations:** Naval Health Research Center, San Diego, CA; Leidos, Inc., San Diego

Recurring headache, a broad term that includes chronic migraine as well as other headache diagnoses, is a major cause of lost duty time among U.S. military women. ^1^ Migraine, in particular, is as much as 3 times more prevalent among women and is the headache type most affected by changes in estrogen levels that may result from biological processes (e.g., menopause, pregnancy) or use of exogenous hormones (e.g., hormonal contraceptives). ^2^

Although prior studies have compared recurring headache among male and female service members, ^3 - 5^ few have focused on the association of recurring headache with women's health issues. ^6^ Furthermore, the availability of treatments for certain headache diagnoses, such as use of contraceptives to treat migraine without aura, suggests that studies of recurring headache and women's health issues could inform targeted health care strategies. ^7^

This cross-sectional study of self-reported “recurring headaches/migraines,” referred to in this report as “recurring headache,” focused on 2 specific aims: 1) examining univariate associations of recurring headache with demographics and women's health characteristics and 2) examining age-specific associations of recurring headache with menstrual-related issues.

## Methods

Data for this cross-sectional study were drawn from the 2021 Periodic Health Assessment (PHA). ^8^ The PHA is a standardized, annual health assessment for all military services that assesses individuals' medical readiness. The PHA is comprehensive and collects data on survey items related to chronic medical conditions such as recurring headache and women's health issues.

The PHA Data Sharing Agreement restricted analyses to U.S. Navy and Marine Corps personnel through 2021. Because a new version of the PHA questionnaire was introduced mid-2021, assessments in this study included those completed from August through December 2021. The main outcome was recorded as a closed question prompt on the PHA that asks for self-reported experience of “recurring headaches/migraines” within the prior year. The PHA asks survey participants, “Since your last PHA, have you experienced any of the following health conditions that either required medical care or impacted your duty performance (or both) and if so, what is your status?”

To examine recurring headache status (regardless of medical care or performance), we dichotomized answers to ‘yes’ or ‘no.’ Women's health variables of interest were hypothesized determinants (or surrogates for determinants) of recurring headache, reflecting putative relevance to estrogen-associated migraine: pregnancy history, contraceptive methods, history of total hysterectomy (as a surrogate for oophorectomy), post-menopausal status, and menstrual-related issues. ^2^ Women's health questions and possible answers from the PHA are displayed in **Table 1** . Demographics included age (in years), pay grade (% enlisted), number of deployments (% >= 1), service branch (% Marine Corps or Navy), service component (% active duty or reserve), and “temporary profile or temporary limited duty” (LIMDU/TLD) status (% ‘yes’).

**TABLE 1.** Distribution of Demographic and Women's Health Characteristics of Respondents, With and Without Self-Reported Recurring Headaches or Migraine, Annual Periodic Health Assessment, Female U.S. Navy and Marine Corps Service Members, 2021

Characteristics	Recurring Headaches or Migraine				*P* -value ^a^
	No (n=13,570)		Yes (n=4,059)		
	No.	%	No.	%	
Age, y ^b^					<0.0001
18–24	5,324	39.2	1,008	24.8	
25–29	3,278	24.2	968	23.8	
30–34	2,159	15.9	786	19.4	
35–39	1,446	10.7	635	15.6	
40 +	1,360	10.0	662	16.3	
Pay grade					<0.0001
Enlisted	10,348	76.3	3,456	85.1	
Deployments, n					<0.0001
>=1	4,143	30.5	1,507	37.1	
Service branch					0.7785
Marine Corps	2,418	17.8	716	17.6	
Navy	11,152	82.2	3,343	82.4	
Component					<0.0001
Active	10,906	80.4	3,501	86.3	
Reserves	2,658	19.6	558	13.7	
Temporary profile or LIMDU/TLD					<0.0001
Yes	1,066	7.9	674	16.6	
“Which of the following best describes you?”					<0.0001
I am or may be pregnant	645	4.8	240	5.9	
I was pregnant or just delivered within the past 6 months	609	4.5	217	5.3	
I was pregnant or delivered 6-12 months ago	423	3.1	198	4.9	
I am not pregnant now, and was not pregnant or delivered in the past 12 months 11,893	87.6	3,404	83.9		
“Since your last PHA, what contraceptive methods, if any, have you and your partner(s) been using to prevent pregnancy? Mark all that apply”					
Long term IUD (including copper or progesterone) or implant, yes	3,231	23.8	894	22.0	0.0459
Injectable—every 3 months, yes	200	1.5	63	1.6	0.9229
Daily—birth control pills, yes	1,937	14.3	493	12.1	0.0022
Monthly—contraceptive patch/vaginal ring, yes	296	2.2	111	2.7	0.1171
Emergency contraception (such as Plan B), yes	481	3.5	126	3.1	0.3879
“Have you had a total hysterectomy (uterus and cervix removed)?”					<0.0001
Yes	161	1.2	132	3.3	
**“Are you postmenopausal and no longer experiencing menstrual cycles?”**					<0.0001
Yes	457	3.4	156	3.8	
“Do you have heavy and/or irregular menstrual cycles/pain or premenstrual syndrome (PMS)?”					<0.0001
Yes, but I am in treatment and having no problems	766	5.6	313	7.7	
Yes, and I am having ongoing issues	2,119	15.6	1,359	33.5	
No	10,150	74.8	2,150	53.0	
Missing	535	3.9	237	5.8	

Abbreviations: n, number; No., number; y, years; LIMDU, limited duty; TLD, temporary limited duty; PHA, Periodic Health Assessment; IUD, intrauterine device; PMS, pre-menstrual syndrome.

a *P* -values are for the χ ^2^ test of independence for categorical variables.

b Values of age among respondents with no recurring headache do not sum to 100% because 3 respondents were age 17 years.

To examine distributions of demographics for women's health characteristics—Aim 1—we displayed percentages among women with or without self-reported recurring headache. *P* -values were computed from t-tests or Chi-square tests. For Aim 2, to examine age-specific associations of recurring headache with menstrual-related issues (i.e., responding ‘yes’, or endorsing heavy and/or irregular menstrual cycles/pain or pre-menstrual syndrome), we used log-binomial regression to test interaction terms for statistical significance, defined as *p* <0.05. Estimates were stratified into 4 age groups: 18–29, 30–39, 40–49, and 50–64 years. Age-specific prevalence ratios (PRs) and 95% confidence intervals (CIs) were estimated from log-binomial regression of the probability of recurring headache.

## Results

Overall, 17,629 women who completed the 2021 PHA were included in this study. The prevalence of self-reported recurring headache was 23.0%. **Table 1** demographics show that women with self-reported recurring headache were more likely than women without self-reported recurring headache to be older, enlisted, deployed at least once, active duty, or on LIMDU/TLD. Associations with women's health variables suggest that those with recurring headache, compared to those without, were more likely to endorse “[are] or may be pregnant,” history of total hysterectomy, or post-menopausal status. The occurrence of menstrual-related issues was strongly associated with recurring headache, particularly among those who endorsed ongoing issues. Notably, univariate associations showed that women with recurring headache were less likely to report using a long-term intrauterine device (IUD) (22.0% vs. 23.8%, *p* =0.0459) or daily birth control pills (12.1% vs. 14.3%, *p* =0.0022).

As shown in **Table 2** , age-specific associations of recurring headache with menstrual-related issues were stronger among women in the younger age groups, particularly those who endorsed ongoing issues. *P* -values for each interaction term of age (as a continuous covariate) and menstrual-related issues (“Yes, but in treatment and no issues” or “Yes, but having ongoing issues”) were *p* =0.6313 and *p* =0.0711, respectively. PRs and 95% CIs of recurring headache among women with ongoing menstrual-related issues (compared with no issues) were 2.4 (2.2, 2.6); 2.3 (2.1, 2.5); 1.7 (1.5, 2.0); and 3.1 (1.4, 7.0)—among women ages 18–29, 30–39, 40–49, and 50–64 years, respectively. Among women 50–64 years of age, wider CIs likely reflected a smaller sample in this age group.

**TABLE 2.** Age-Specific Prevalence and Prevalence Ratios of Self-Reported Recurring Headaches or Migraines, by “Heavy and/or Irregular Menstrual Cycles/Pain or Premenstrual Syndrome,” Annual Periodic Health Assessment, Female U.S. Navy and Marine Corps Service Members, 2021

Age Group	“Heavy or Irregular Menstrual Cycles, Pain, or PMS?” ^a^	Total (n)	Recurring Headache or Migraine Cases (n)	Prevalence (%)	Prevalence Ratio ^b^	95% CI
18–29	No	7,592	1,071	14.1	1.0	Reference
	Yes, but in treatment and no issues	629	145	23.1	1.6	1.4–1.9
	Yes, but having ongoing issues	2,208	733	33.2	2.4	2.2–2.6
30–39	No	3,635	787	21.7	1.0	Reference
	Yes, but in treatment and no issues	320	120	37.5	1.7	1.5–2.0
	Yes, but having ongoing issues	943	467	49.5	2.3	2.1–2.5
40–49	No	1,003	283	28.2	1.0	Reference
	Yes, but in treatment and no issues	117	45	38.5	1.4	1.1–1.7
	Yes, but having ongoing issues	308	151	49.0	1.7	1.5–2.0
50–64	No	67	9	13.4	1.0	Reference
	Yes, but in treatment and no issues	13	3	23.1	1.7	0.5–5.5
	Yes, but having ongoing issues	19	8	42.1	3.1	1.4–7.0

Abbreviations: n, number; PMS, pre-menstrual syndrome; CI, confidence interval.

a Service members with missing responses to “heavy and/or irregular menstrual cycles/pain or premenstrual syndrome” are excluded from table totals.

b Prevalence ratios and 95% CIs are from age group stratified log-binomial regression of the probability of recurring headache.

## Discussion

This study indicated a high prevalence of self-reported recurring headache (23.0%) during a 5-month period in 2021 among U.S. Navy and Marine Corps women. This study's numbers approximate 2011 findings from the Millennium Cohort Study, which included female U.S. active duty, reserve, and Guard members (n=12,409), and reported provider-diagnosed migraine or recurrent severe headache occurrence within the past year among 20.9% or 22.3% of military women, respectively. ^9^ These estimates are higher than the female general population's annual prevalence (17%) ^10^ but lower than lifetime migraine prevalence (30.1%) among female veterans. ^3^

Although this study could not differentiate between specific headache diagnoses, noted associations with estrogen-related health characteristics suggest that a substantial proportion of women may be at risk for estrogen-associated migraines upon clinical evaluation. ^2^ This cross-sectional study could not establish temporal relationships between variables of interest, and our findings have limited ability to support causal inference. Nevertheless, the lower prevalence of recurring headache among women in treatment for menstrual-related issues warrants consideration of whether individuals reporting ongoing menstrual-related issues could benefit from hormonal contraception or other hormone-related treatments of estrogen-associated headache, ^11^ which is consistent with the literature. ^2 , 12^

This work adds to the literature on recurring headache among women in the U.S. Navy and Marine Corps. Limitations of this preliminary study include an inability to differentiate between diagnostic subtypes of headache, absence of covariates of interest not recorded by the PHA, and cross-sectional analysis that precludes causal inference. Strengths of this work include its large sample size and estimation of age-specific prevalences. Additional work is needed to understand patterns of headache and migraine among U.S. military women, but this study highlights the importance and relevance of women's health issues in female service members with recurring headache.
